# Somatostatin Containing δ-Cell Number Is Reduced in Type-2 Diabetes

**DOI:** 10.3390/ijms24043449

**Published:** 2023-02-09

**Authors:** Lakshmi Kothegala, Caroline Miranda, Meetu Singh, Jean-Philippe Krieger, Nikhil R. Gandasi

**Affiliations:** 1 Cell Metabolism Lab (GA-08), Department of Developmental Biology and Genetics (DBG), Indian Institute of Science (IISc), Bengaluru 560012, India; 2 Department of Metabolic Physiology, Institute of Neuroscience and Physiology, University of Gothenburg, Box 430, 40530 Gothenburg, Sweden; 3 Department of Medical Cell Biology, Uppsala University, BMC 571, 75123 Uppsala, Sweden

**Keywords:** Type-2-Diabetes, islet-hormone secretion, multi-photon imaging, Confocal microscopy, pancreatic islets

## Abstract

Recent developments suggest that increased glucagon and decreased somatostatin secretion from the pancreas contribute to hyperglycaemia in type-2 diabetes (T2D) patients. There is a huge need to understand changes in glucagon and somatostatin secretion to develop potential anti-diabetic drugs. To further describe the role of somatostatin in the pathogenesis of T2D, reliable means to detect islet δ-cells and somatostatin secretion are necessary. In this study, we first tested currently available anti-somatostatin antibodies against a mouse model that fluorescently labels δ-cells. We found that these antibodies only label 10–15% of the fluorescently labelled δ-cells in pancreatic islets. We further tested six antibodies (newly developed) that can label both somatostatin 14 (SST14) and 28 (SST28) and found that four of them were able to detect above 70% of the fluorescent cells in the transgenic islets. This is quite efficient compared to the commercially available antibodies. Using one of these antibodies (SST10G5), we compared the cytoarchitecture of mouse and human pancreatic islets and found fewer δ-cells in the periphery of human islets. Interestingly, the δ-cell number was also reduced in islets from T2D donors compared to non-diabetic donors. Finally, with the aim to measure SST secretion from pancreatic islets, one of the candidate antibodies was used to develop a direct-ELISA-based SST assay. Using this novel assay, we could detect SST secretion under low and high glucose conditions from the pancreatic islets, both in mice and humans. Overall, using antibody-based tools provided by Mercodia AB, our study indicates reduced δ-cell numbers and SST secretion in diabetic islets.

## 1. Introduction

A well-functioning pancreas preserves glucose homeostasis and hence prevents diabetes. Most of the work related to maintenance of glucose homeostasis has been focused on insulin secretion. Recently, the contribution of increased levels of circulating glucagon and decreased levels of somatostatin in type 1 and type 2 diabetes has been recognized as a contributor to hyperglycemia [1,2,3].

**Figure 1:**
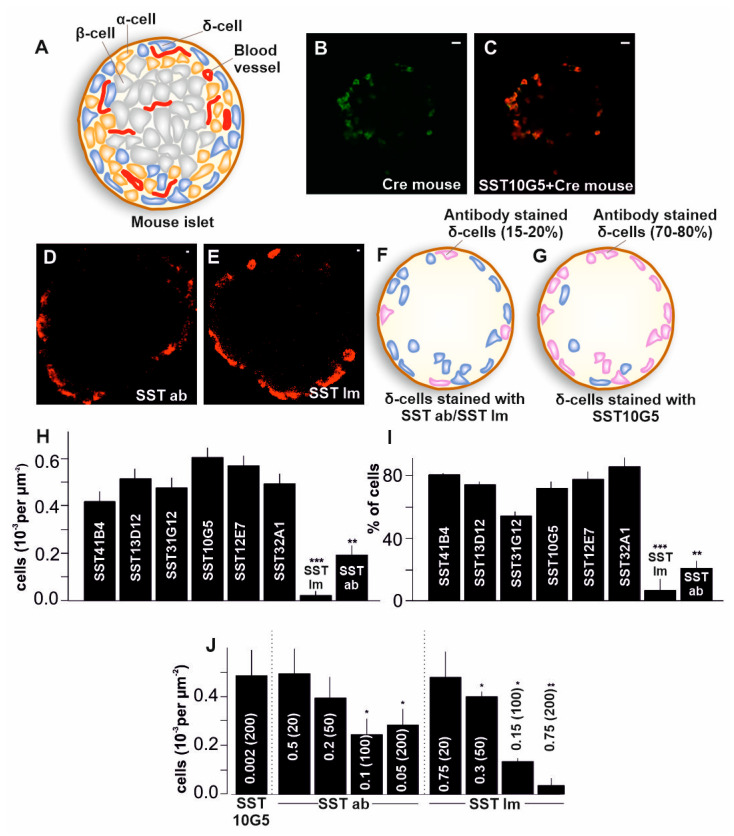
Co-localization of immune-stained δ-cells with transgenic islets—(**A**) Cartoon depiction of a mouse islet showing the α, β and δ-cells, (**B**) View of islet from SST-iCre mice with δ-cells represented in green (Scale bar = 10 µm), (**C**) Islets from SST-iCre mice labelled with δ-cell specific antibody SST10G5 (Scale bar = 10 µm), (**D**) Islets from SST-iCre mice labelled with SST-14 specific commercial antibody from Abcam (SST ab) (Scale bar = 1 µm), (**E**) Islets from SST-iCre mice labelled with SST-14/28 specific commercial antibody from Immunostar (SST Im) (Scale bar = 1 µm), (**F**) Cartoon depiction of δ-cells stained with commercial antibodies (SST ab/SST Im), (**G**) Cartoon depiction of δ-cells stained with newly developed antibodies (SST10G5), (**H**) Total number of δ-cells stained with SST antibodies in transgenic mice with eYFP label expressed on SST-Cre promotor. Each experiment was repeated on 5–10 islets on two different days using 3–6 mice, (**I**) Co-localization of antibody labelled cells with eYFP expressing cells specific for SST-Cre promotor. Each experiment was repeated on 6–8 islets on two different days using 5–8 mice, (**J**) Number of SST-cells stained with different dilutions of SST antibodies. Total number of antibody stained δ-cells with different dilutions of antibodies specified in the figure (20, 50, 100 and 200 are dilutions of 1:20, 1:50, 1:100 and 1:200 respectively). Each experiment was repeated on 8–10 islets on two different days using 3–5 mice. (* *p* < 0.05, ** *p* < 0.01, *** *p* < 0.001).

Islets contain β-cells secreting insulin, α-cells secreting glucagon, and δ-cells secreting somatostatin. In rodent islets, β-cells form the core surrounded by peripheral distribution of α- and δ- cells (Figure 1A) [4]. Islets obtained from human donors have a differential distribution of cells compared to mouse islets. Mouse islets have approximately 70% β-cells, 20% α-cells, and 10% δ-cells [5]. The distribution of human islets came to light only a decade ago, showing that they do not have anatomical subdivisions. β-, α-, and δ- cells are scattered throughout the human islet and β-cells although few associate with other endocrine cells in the islet [5]. Interaction between islet cells happens via cell–cell adhesion between similar cells or dissimilar cells in a paracrine manner. Cell–cell adhesion-based interaction with dissimilar cells can be observed between α- and β-cells, α- and δ-cells, or β- and δ-cells [6]. The δ-cells have specialized structures which are called projections or filopodia; these form the basis of contact with neighboring α-and β-cells, regulating the secretion from these cells, and thus maintaining the glucose homeostasis [7].

Initially, Somatostatin (SST) was discovered in hypothalamus in the form of SST14 in 1973 [8]. Soon, a very similar peptide was isolated in the gut, which was later identified as SST28. SST28 is mostly confined to the intestinal duodenal mucosal cells and SST14 to stomach, pancreatic δ-cells, and neurons [9]. Both SST14 and SST28 arise from a common prosomatostatin which is encoded by a single gene after the posttranslational cleavage [10]. SST content in gut, hypothalamus, and islet cells is regulated by post-translational mechanisms, such as proteolytic cleavage of pre-prosomatostatin, phosphorylation, and sumoylation. The pre-translational modifications, such as methylations, polymorphisms, and regulation of transcription factor activity, also regulate the SST content [11].

The secretion of SST and other islet hormones has been measured using the radioimmunoassay (RIA) for many decades. The RIA based assays for islet hormones insulin, glucagon, and somatostatin were considered as a reliable method to measure the physiological concentrations of these hormones [12]. This changed a few years later with efficient ELISA assays available for the measurement of insulin [13] and glucagon [14,15] respectively. The RIA remained an efficient method for the measurement of SST until very recently [16] due to the non-availability of efficient ELISA assays. RIA assays bring radioactivity risks. The requirement of special permissions and licenses has led to the decreased usage of RIA assays as most of labs do not have permission to carry out such assays [17]. Hence, we developed an ELISA assay that could efficiently detect SST secreted from isolated islets. For this purpose, the efficiency of commercially available antibodies to label δ-cells in specialized transgenic mouse models with eYFP labelled δ-cells was assessed. We found that the commercially available antibodies were not able to detect all the δ-cells in an islet. Hence, we thank Mercodia AB for the kind gift of the six antibodies that can detect both SST14 and SST28 (details of the antibodies in Table 1), thus providing access to the entire δ-cell population in the islet. We evaluated the efficiency of these antibodies by immunostaining islets from mice expressing eYFP specifically in δ-cells, islets from healthy and T2D human donors. These antibodies showed high efficiency as they detected the majority of the eYFP labelled δ-cells. These antibodies were able to detect somatostatin secreting cells in sections of hypothalamus and gut tissues, confirming their specificity for SST. These antibodies were used to test a novel SST assay which can measure SST secreted from the mouse and human islets at different concentrations of glucose. The antibodies were also used to study the arrangement of δ-cells that differed between mouse and human islets [5]. Overall, our study presents antibodies that are highly efficient in detecting δ-cells and measuring SST secreted from δ-cells. Further, the study gave insights about the changes in the δ-cell number and expression of SST in human islets during type-2 diabetes.

## 2. Results

### 2.1. Co-Localization of Immuno-Stained Cells in Transgenic Islets 

To find an efficient way to label somatostatin secreting δ-cells in the islets, we assessed the commercially available antibodies targeted against SST14 and SST28. We utilized a mouse model, the ChR2^+/−^-SST^+/−^iCre (SST-iCre) C57BL/6J mouse, which has an SST specific promotor that labels the δ-cells with eYFP (Figure 1B–E). We found that all the δ-cells in SST-iCre mice which were fluorescently labelled with eYFP were not labelled with commercially available antibodies (Figure 1F,H). Therefore, we obtained six antibodies from Mercodia AB, targeted at somatostatin (see Table 1 for details) and tested these antibodies in SST-iCre mice. The antibodies we tested were SST41B4, SST13D12, SST31G12, SST10G5, SST12E7, and SST32A1. Their efficiency to label all the δ-cells in an islet was compared to the commercially available control antibodies (SST Im and SST ab). The results showed a higher degree of overlap over eYFP labelled δ-cells with the antibodies from Mercodia AB (Figure 1C,G–I). The total number of antibody-labelled δ-cells was counted (Figure 1H), which showed a significantly higher number of δ-cells with these antibodies compared to the commercially available antibodies. All the antibodies showed more than 70% co-localization with eYFP labelled cells, except SST31G12 which showed around 50% co-localization and CTRL antibodies which showed a very low degree of co-localization (<20%, Figure 1I). 

**Figure 2:**
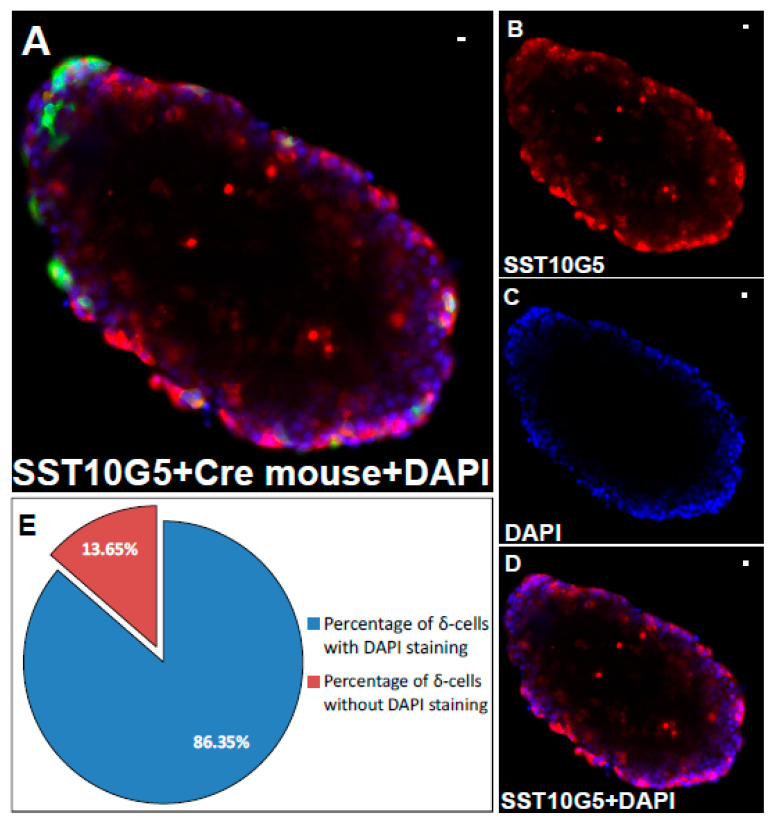
Immunostained δ-cells counterstained with nuclear stain DAPI—(**A**) Islets from SST-iCre mice labelled with δ-cell specific antibody SST10G5 counterstained with nuclear stain DAPI (Scale bar = 10 µm), (**B**) Immunostained islets in (**A**) showing the SST10G5 δ-cell labelling (Scale bar = 10 µm), (**C**) Immunostained islets in (**A**) showing the DAPI staining (Scale bar = 10 µm), (**D**) Immunostained islets in (**A**) showing the merged image with SST10G5 and DAPI (Scale bar = 10 µm), (**E**) Pie chart showing the quantification of the SST10G5 δ-cells labelled cells conterstained with DAPI nuclear stain. (The SST10G5 antibody is in red, DAPI is in blue, transgenically labelled δ-cells in green.).

Next, we tested different dilutions (1:20, 1:50, 1:100, and 1:200) for the commercially available antibodies. This translates to concentrations of 0.5, 0.2, 0.1 and 0.05 mg/mL for SST14 specific commercially available antibody (SST ab) and 0.75, 0.3, 0.15, and 0.075 mg/mL for SST14/28 commercially available antibody (SST Im), respectively. These concentrations were compared to SST10G5 antibody at 0.002 mg/mL (1:200 dilution), which was able to label all the δ-cells in SST-iCre mice. We found that the SST14 or SST14/28 commercially available antibodies showed almost the same degree of co-localization compared to the Mercodia AB antibodies at dilutions below 1:20 (Figure 1J). Neither of the commercially available antibodies were as efficient at higher dilutions compared to the high specificity antibodies from Mercodia AB (such as SST10G5), which was efficient at 1:200 dilution. This gave a basis to establish the working concentrations for all the antibodies tested here. These results confirm that the efficiency of the tested antibodies surpasses the commercially available antibodies. To visualize the localization of the cells within an islet, and to check their viability, the mouse islets were immunostained with SST10G5 and a nuclear counterstain, DAPI (Figure 2A–D). The quantification suggested that more than 85% of the immunostained δ-cells showed a positive DAPI staining (Figure 2E).

**Figure 3:**
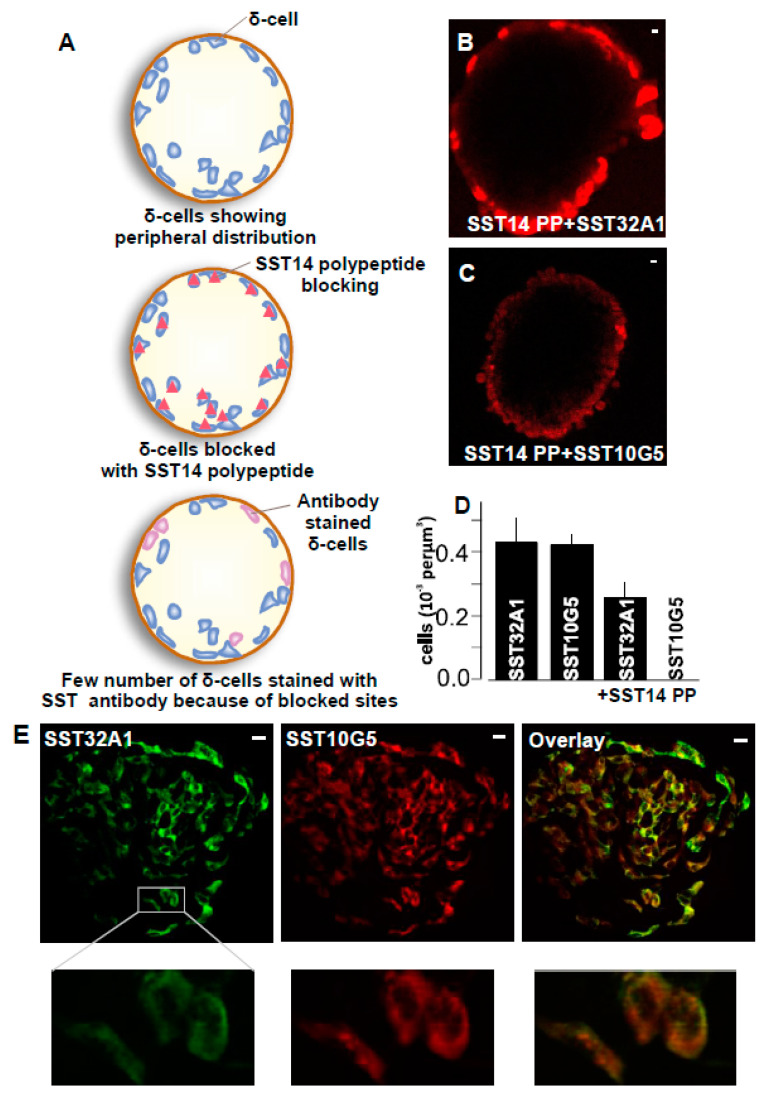
Both SST-14 and SST-28 co-express in mouse islets—(**A**) Cartoon representation for blocking of islets with SST14 polypeptide (SST14 PP), (**B**) Images for islets pre-incubated with SST14 PP prior to staining with SST32A1 antibody (Scale bar = 1 µm), (**C**) Images for islets pre-incubated with SST14 PP prior to staining with SST10G5 antibody (Scale bar = 1 µm), (In (**B**,**C**), the SST10G5 and SST32A1 immunostaining are in red.) (**D**) Number of cells per islet from the experiments as displayed in (**A**,**B**) in comparison with controls where islets were stained with SST10G5 or SST32A1 without pre-incubation with SST14 polypeptide. Each experiment was repeated on at least 7 islets on two different days using 3–5 mice, (**E**) Islets from mice labelled with SST10G5 that mostly detects SST14 and SST32A1 which mostly detects SST28 and overlays. Zoomed images of single δ-cells from each of the conditions specified on the left side of the image (Scale bar = 10 µm). (SST32A1 immunostaining is in green, SST10G5 is in red).

We used SST14 polypeptide (SST14 PP) to block the islets before staining, to test the specificity of the antibodies obtained from Mercodia AB for SST14 such as SST10G5 and for SST14/28 such as SST32A1 respectively (Figure 3A). These were compared with controls where no such blocking was performed with SST14 polypeptide. The islets stained with SST32A1 in the presence of SST14 polypeptide were visualized with δ-cells whereas the islet stained with SST10G5 in the presence of SST14 polypeptide had very few labelled δ-cells (Figure 3B,C). Analysis of many such islet images for the number of stained δ-cells and comparison with control without SST14 polypeptide showed fewer δ-cells with SST32A1 but virtually no δ-cells with SST10G5 (Figure 3D). This was despite most δ-cells in the SST-iCre mice stained with eYFP overlapping with SST10G5. The results show that the SST14 polypeptide blocks sites completely for SST10G5 which mostly detects SST14, but blocks sites partially for SST32A1 which detects both SST14 and SST28. Many islets were analyzed (Figure 3E) showing that both SST14 and SST28 co-occur in the δ-cells of mouse pancreas. This is in line with evidence suggesting that SST14 and SST28 co-express in pancreatic δ-cells [18].

### 2.2. SST-14 vs. SST-28 Dilemma 

**Figure 4:**
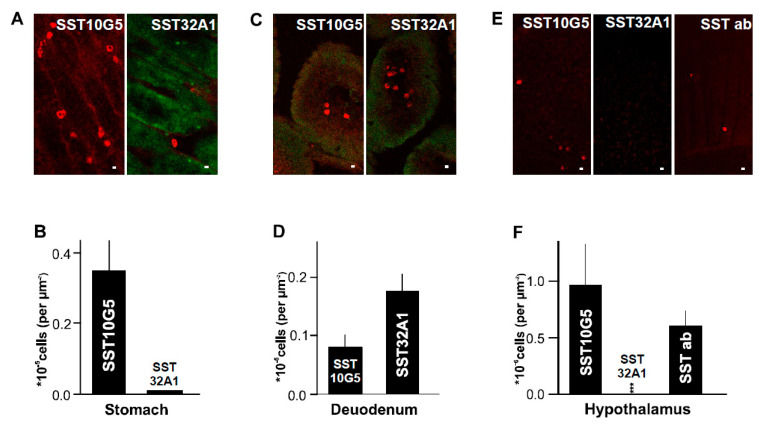
SST-14 expression in the hypothalamus and gut tissues—(**A**) Images showing a cryo-section of mouse stomach stained with SST10G5 or SST32A1 (Scale bar = 1 µm), (**B**) Number of cells in image (**A**) labelled with SST10G5 or SST32A1 in the stomach tissues. Each experiment was repeated on two different days using 3–4 mice. At least 6 sections were analysed in each case, (**C**) Image showing a cryo-section of mouse duodenum stained with SST10G5 or SST32A1 (Scale bar = 1 µm), (**D**) Number of cells in image (**C**) labelled with SST32A1 or SST10G5 in the duodenal tissues. Each experiment was repeated on two different days using 3–4 mice. At least 6 sections were analysed in each case, (**E**) Image showing a cryo-section of mouse hypothalamus stained with SST10G5 or SST32A1 or commercialy available SST14 antibody (SST ab) (Scale bar = 1 µm), (**F**) Number of cells in image (**E**) labelled with SST10G5, SST32A1 or commercially available SST14 antibody (SST ab) in the hypothalamus. Each experiment was repeated on two different days using 5–6 mice. At least 5 sections were analysed in each case. (*** *p* < 0.001).

SST-producing cells are mostly distributed in the nervous system and the gut, apart from the pancreas [19]. SST in gut is confined to submucous and myenteric plexus regions. To evaluate the distribution of SST in gut cells, we stained longitudinal sections of stomach and duodenum with SST10G5 and SST32A1 antibodies (Figure 4A–D). We found that SST10G5 antibody stained more enteroendocrine cells containing SST in the stomach (Figure 4A,B). In comparison, SST32A1 antibody which mostly stains SST28, stained more cells in the duodenum (Figure 4C,D). A small number of cells positive for SST10G5 or SST32A1 were found both in stomach and intestine (duodenum). The cells stained with SST10G5 were half the number of cells stained with SST32A1 in the duodenum, whereas almost no cells stained with SST32A1 were found in the stomach. The results suggest that the duodenum has both SST14 and SST28 containing cells whereas the stomach probably has more SST14 cells. This confirms the previous observations suggesting that SST28 is localized to intestinal mucosa cells, whereas SST14 is mostly found in the gastric parts of the gut [9].

In the brain, 80% of the SST immunoreactivity is observed in the hypothalamus [20]. Therefore, we obtained cryo-sections of mouse hypothalamus. The hypothalamus sections were stained with SST10G5 antibody which mostly detects SST14, SST32A1 which mostly detects SST28, and some SST14 specific commercially available antibodies. SST-positive cells could be observed in sections stained with SST10G5 and commercially available SST14 antibody but not with SST32A1 in the hypothalamus sections (Figure 4E). Quantification of the images showed a greater number of cells with SST10G5 compared to commercially available SST14 antibody but no stained cells with SST32A1 (Figure 4F). The results confirm that SST14 is predominantly present in the brain and SST10G5 is the most efficient antibody in detecting the same. Overall, the antibodies that we tested have higher specificity in gut and hypothalamus tissues for both SST14 and SST28, thus demonstrating wider importance.

### 2.3. Distribution of δ-Cells in Mouse Compared to Human Islet 

**Figure 5:**
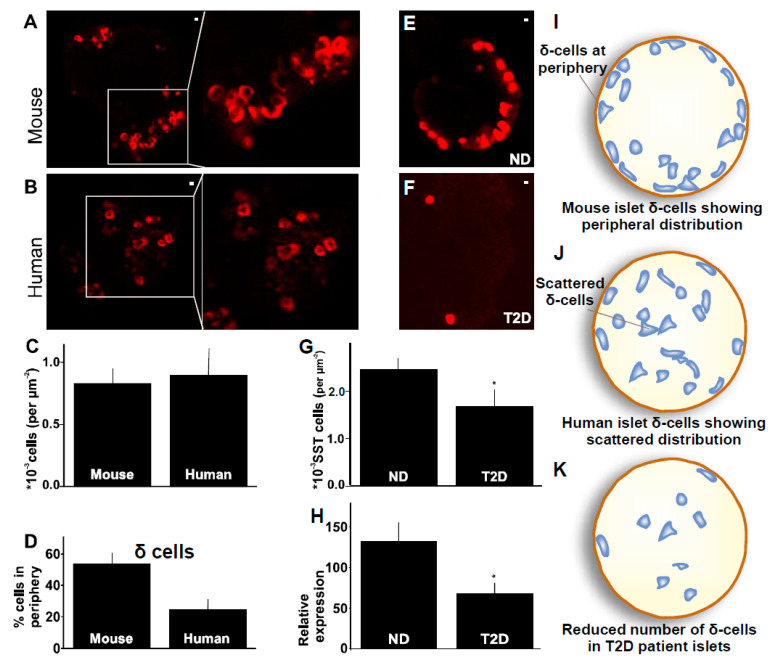
Distribution of δ-cells in mouse compared to human islet, how this distribution is affected during human T2D—(**A**) A mouse islet stained with SST antibody. Inset shows peripherally distributed δ-cells (Scale bar = 1 µm), (**B**) A human islet stained with SST antibody. Inset shows scattered δ-cells (Scale bar = 1 µm), (**C**) Total number of δ-cells in an islet. Each experiment was repeated on at least 5 islets on two different days using 2–4 mice or 3 donor islets respectively, (**D**) Percentage of cells distributed in the periphery of the islet. Each experiment was repeated on at least 5 islets on two different days using 2–4 mice or 3 donor islets respectively, (**E**) Distribution of δ-cells in an islet from non-diabetic donor (Scale bar = 1 µm), (**F**) Distribution of δ-cells in an islet from T2D donor (Scale bar = 1 µm), (**G**) Total number of δ-cells per islets from T2D or non-diabetic donors. Each experiment was repeated on at least 5 islets using 3 donor islets (received on sperate days) for ND or T2D respectively, (**H**) Relative expression of SST in islet tissue in comparison with housekeeping genes in non-diabetic and T2D donor islets. Each experiment was repeated on at least 5 islets using 3 donor islets (received on sperate days) each for ND or T2D respectively, (**I**) Cartoon representation for peripheral distribution of δ-cells in mouse islets, (**J**) Cartoon representation for scattered distribution of δ-cells in human islets, (**K**) Cartoon representation for reduced number of δ-cells in T2D patient islets. (* *p* < 0.05).

The distribution of cells in the human islets being different from murine islets has been documented for over a decade [5]. We wanted to evaluate the distribution of specifically δ-cells using the antibody described in previous sections. To evaluate the distribution and interspecies discrepancy in these islets, islets obtained from mice or human donors were stained with the SST10G5 (Figure 5A,B). The immunofluorescence staining showed that 50% of δ-cells are localized close to the periphery of the islet in the mouse, whereas the same number in human islets corresponds to only 30% (Figure 5D). Peripheral cells are identified as cells localized just within the capsule, the limiting layer after which no further fluorescence can be detected in the images. These changes in the distribution of δ-cells in an islet did not impact the total number of δ-cells in an islet (Figure 5C). The results confirm earlier observations of the δ-cell distribution in mouse and human. The δ-cells are mostly peripherally distributed in mouse compared to human islets where no such polar distribution pattern exists (Figure 5I,J) [5].

### 2.4. δ-Cells in Human Type-2 Diabetes 

The distribution of β- and α-cells has been extensively investigated and documented in non-diabetic islets compared to the T2D diabetic islets [21] but the distribution of δ-cells has not been studied in the T2D. Here, we investigate the δ-cell distribution with δ-cell specific antibody SST10G5 in non-diabetic and T2D human islets (Figure 5E,F). The δ-cells were counted and normalized according to the area of the islet. A significant reduction in the number of δ-cells in T2D islets was observed as compared to the non-diabetic islets (Figure 5G,J,K). This coincides with a decrease in peripherally distributed δ-cells which are significantly greater in number in non-diabetic human islets. To verify if the fewer δ-cells in human T2D islets is due to the reduced expression of somatostatin, we assessed mRNA expression. The somatostatin expression was significantly decreased as well in the human T2D islets (Figure 5H) consistent with previous observations [21]. This confirms that the decreased somatostatin expression contributes to fewer δ-cells in human T2D islets.

### 2.5. A Novel Somatostatin Assay

**Figure 6:**
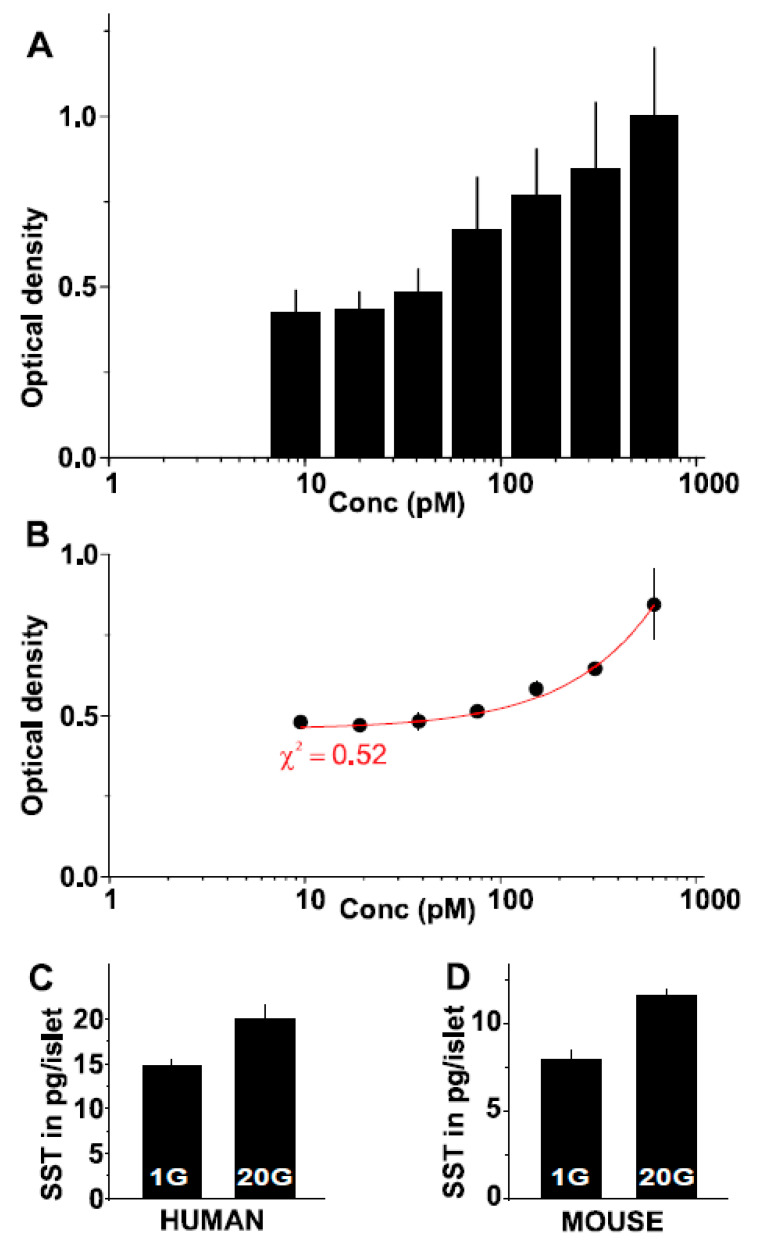
Assay to detect SST secretion with varying glucose concentrations—(**A**) Optical density plotted against the concentration of SST diluted to prepare standards, (**B**) Same as (**A**) but fitted with exponential decay function with chi-square value of 0.52, (**C**) SST secretion interpolated against the curve from human islets treated with 1 and 20 mM glucose (1G and 20G groups respectively). Each experiment was repeated with 3 replicates using 3 donor islets (received on sperate days), (**D**) SST secretion interpolated against the curve from mouse islets treated with 1 and 20 mM glucose (1G and 20G groups respectively). Each experiment was repeated with 3 replicates using on two different days using 3–6 mice.

Based on the immunostaining data in Figure 1H, we selected SST10G5 as the antibody of choice for the development of a somatostatin assay that can detect standards and somatostatin released from mouse and human islets. SST-14 peptide was used as a standard here. Dilutions were made between 9.5 pM to 305 nM, serially resulting in seven different concentrations as shown in Figure 6A,B. SST10G5 was used as a primary antibody on the somatostatin coated plates and anti-mouse conjugated HRP antibody was used as secondary antibody for a direct ELISA based technique. The plate was read as described in the methods to obtain a uniform increase in the optical density based on the increasing concentrations (Figure 6A). This was repeated multiple times before the averages were fitted using an exponential decay function with chi-square value of 0.52 (Figure 6B). Somatostatin secretion for the islet spent culture media of mouse and human samples treated with 1 mM glucose (1G group) and 20 mM glucose (20G group) was measured in parallel with the standards (see methods). Somatostatin secretion was significantly higher in 20 mM glucose compared to secretion from 1 mM glucose in both the human and mouse islet spent culture media respectively (Figure 6C,D). When samples were interpolated against the curve, the 20G group showed a 25% increase in measured SST-14 for human islets (healthy donors) and a 30% increase in mouse islets. Overall, we developed a reliable somatostatin assay that can measure somatostatin secreted from both mouse and human islets.

## 3. Discussion 

Most currently available treatment strategies for T2D potentiate insulin secretion (the sledgehammer approach), completely ignoring the actions of glucagon and somatostatin. The resulting abnormal levels of insulin in the blood are not counteracted because the other hormones are also lacking in the diabetic environment [22]. This condition results in the death of 10% of diabetic patients treated with insulin [23]. Earlier studies emphasize a reduction in the insulin levels in T2D patients [24]. Recent studies have highlighted the importance of the multi-hormonal homeostasis in the islet environment and not only insulin. Moreover, landmark studies showed that glucagon secretion and upstream mechanisms are affected in T2D [25,26] and while there are only a few studies on the secretion of somatostatin, they have highlighted the significance of the paracrine regulation that δ-cells demonstrate within islets [7,25]. Previous studies highlight the reduction in the number of β-cells during T2D [27]. Limited studies on δ-cells might be due to the small percentage they represent within the islet, making it difficult for them to be detected in single cell assays. Studies on secretion of SST from whole islets are also limited by lack of reliable assays to accurately measure somatostatin secretion. 

Most of the current studies on the detection of somatostatin secreted from the islets have relied on radio-labelled assays (RIA) [12,16]. These assays have high radioactivity risks and most labs do not have permission to carry out such assays [17]. There are SST ELISA assays available on the market, one of which is a competitive ELISA assay used for the detection of SST between concentrations of 0.13 and 1.16 pg/mL. This assay has been used in the detection of SST in the plasma [15], intestine cultures [28], isolated islets [29] and SST secreting cell lines [30]. However, this assay is only able to measure total SST content from the islets or islet cells, not SST secreted from the pancreas. With the thought of developing a novel ELISA to detect secreted SST, we initially tested antibodies of SST that are part of commercially available SST assays and found that they have to be used under very high concentrations to efficiently detect all the δ-cells in an islet (Figure 1E). The antibodies we obtained from Mercodia AB showed higher efficiency compared to the commercially available antibodies (Figure 1C–E), therefore opening possibilities for developing a new assay. 

Somatostatin producing cells are distributed in high densities in the central and peripheral nervous systems, endocrine pancreas and the gut (reviewed in detail in [31]). In this regard, it was relevant to test the Mercodia AB developed antibodies in all these tissues to see if they can detect SST cells before developing the assay further. Therefore, we assessed if these antibodies were able to detect SST in gut and hypothalamus tissues. SST10G5 was able to detect mostly SST14 in the upper parts of the gut, such as stomach and hypothalamus. SST32A1 was able to mostly detect SST28 in lower parts of the gut. This further confirmed the specificity of these antibodies to detect both SST14 and SST28. This is in accordance with the literature for the hypothalamus [20], gut, and pancreas [9]. Further, there is evidence suggesting that both SST14 and SST28 co-express [18] in pancreatic δ-cells. Our detection of pancreatic δ-cells with both SST10G5 and SST32A1 provides more evidence in this direction, although SST14 might be the predominant form (Figure 3A–C). Most commercially available antibodies are not specific to either SST14 or SST28. The antibodies tested here predominantly detect SST14 or SST28 (details in the Table 1). This is where the ability to detect SST secreting cells in the hypothalamus, gut, and pancreatic tissues gains relevance (Figure 4).

Single-cell islet transcriptomes revealed that SST gene expression is differentially regulated in T2D islets compared to non-diabetes islets [21]. Our results are in line with the transcriptome data (Figure 5H). We further studied how this impacts the δ-cell number. δ-cell number was surprisingly decreased in human T2D, and the decreased number affected mainly the peripheral distribution of δ-cells in the human islet (Figure 5E–G). This is a novel finding in human T2D islets and gives more insight into the changes in δ-cells and overall cytoarchitecture of the islets during type-2 diabetes.

SST plasma levels are altered in the presence of anti-diabetic drugs [15]. Previous assays fail to measure changing concentrations of SST from isolated islets. We believed that this was due to the concentration of SST per islet that can vary between 3–25 pg. Therefore, we successfully exploited antibody SST10G5 to measure SST from mouse and human islets under low (1 mM) and high (20 mM) glucose concentrations. We observed significant changes in SST secreted under these glucose concentrations (Figure 6C,D). Furthermore, this has been the most consistent method of measuring SST secreted from the islets without using radioactive isotopes. Due to high radioactivity risks posed by using radioactive isotopes [17], we believe that the assay proposed will be of huge importance to researchers studying the secretion of islet hormones such as SST in the future.

## 4. Material and Methods

### 4.1. Islet Isolation 

Pancreatic islets were obtained from human cadaveric donors by the Nordic Network for Clinical Islet Transplantation (ethical approval by Uppsala Regional Ethics Board 2006/348) [32] or the ADI Isletcore at the University of Alberta (ethical approval by Alberta Human Research Ethics Board, Pro00001754) [33], with written donor and family consent for use in research. Work with human tissue complied with all relevant ethical regulations for use in research and the study was approved by the Gothenburg Regional Ethics Board, Sweden and ethical committees at Indian Institute of Science, India. Isolated islets were cultured in free-floating sterile dishes in RPMI 1640 culture medium containing 5.5 mmol/L glucose, 10% fetal bovine serum (FBS), streptomycin (100 U/mL), and penicillin (100 U/mL) at 37 °C in an atmosphere of 5% CO_2_ up to a week. 

Mouse islets were isolated from 13 to 17-week-old WT, ChR2^+/−^-SST^+/−^iCre or ChR2^+/−^-Glu^+/−^Cre. Mice were anesthetized and sacrificed by cervical dislocation. Canulation of the bile duct was performed with a 30G needle and a Liberase solution (Liberase TL Roche TM) was injected in the pancreas. The pancreas was excised and digested at 37 °C for 10–12 min. The islets were handpicked under a stereo microscope in Hanks’ balanced salts buffer (HBS) supplemented with 0.1% BSA and 5 mmol/L glucose. Isolated islets were cultured in RPMI 1640 culture medium containing 5.5 mmol/L glucose, 10% fetal bovine serum (FBS), streptomycin (100 U/mL), and penicillin (100 U/mL) at 37 °C in an atmosphere of 5% CO_2_.

All animal experiments were previously approved by the ethics committee at the Sahlgrenska Academy, Gothenburg University, Sweden (approval number: 948/17) and Institutional Animal Ethics Committee (IHEC), Indian Institute of Science, India (approval number: CAF/Ethics/880/2022) respectively.

### 4.2. Immunostaining 

Islets were prepared by tissue fixing with 4% formalin, and permeabilization with 0.3% Triton X in PBS on ice for 30 min. To block, we used PBS supplemented with 5% FBS for 30 min. Somatostatin staining was performed by incubating the islets in a 1:200 dilution of rabbit polyclonal antibody to SST14/28 from ImmunoStar (Cat# 20067, concentration ≈ 15 µg/µL) or rabbit polyclonal antibody against SST14 from AbCam (ab8903, concentration ≈ 10 mg/mL) for 2 h, followed by incubation using Alexa Fluor 594 Donkey anti mouse from Jackson immune laboratories (cat# 715-587-003, 1:200 dilution) for one hour. Somatostatin staining was also conducted with the monoclonal antibodies developed by Mercodia AB, such as SST41B4 (concentration = 0.4 mg/mL), SST13D12 (concentration = 0.8 mg/mL), SST31G12 (concentration = 0.7 mg/mL), SST10G5 (concentration = 0.4 mg/mL), SST12E7 (concentration = 0.7 mg/mL), and SST32A1 (concentration = 0.4 mg/mL) (A kind gift from Mercodia AB). The highest prescribed dilutions recommended for each of these antibodies were followed. They are specified under each experiment in the figure legends. After staining, islets were later washed and mounted on glass slides.

Nuclear staining for supplementary data in SST-iCre mouse islets was performed with DAPI stain and the δ-cells were immunostained with SST10G5. The islets were then washed and mounted on glass slides.

### 4.3. Cryosectioning 

Duodenum, stomach, and hypothalamus tissues were collected and post-fixed in 4% PFA for 2 h, then cryoprotected in 0.1 M phosphate buffer containing 25% sucrose at 4 °C until cryosectioned (10 μm). Sections were obtained after longitudinally embedding the tissue. Cryosections were placed on the same slide in a series to allow direct comparison. The antibody staining for the cryo-sections was performed with protocols described for staining islets above.

### 4.4. Confocal Microscopy 

Confocal microscopy was performed with a Zeiss LSM780 using a 20× and 40× objective (Zeiss) with sequential scanning of the red (excitation 561 nm, emission 578–696 nm), green (excitation 488 nm, emission 493–574 nm), and blue channels (excitation 345 nm, emission 455 nm). Pinhole size was 0.61 μm, corresponding to 1 Airy unit. Images were acquired in 16-bit at gain settings 750 for both the channels. 

### 4.5. Two-Photon Imaging 

Imaging of the stained tissue was performed in a TrimScope II, LavisionBiotec 2-photon microscope using a Ti:Sa laser (MaiTai, Spectra-physics) tuned to 990 nm. Islets were imaged by acquiring z-stacks with 1 μm step-size. Emitted light was separated using a T585LP dichroic mirror into green and red channels and recorded with PMTs-H6780 from Hamamatsu.

### 4.6. Image Analysis 

Images (Figure 1, Figure 2, Figure 3 and Figure 5) were analyzed using Image J. Single images of the islet were counted for number of δ-cells. The area of the islet was calculated using region of interest drawn around the islet. All the counts were normalized according to the area of the islet.

Location of labelled cells in an islet was determined by separating them in to two zones either center or peripheral. The peripheral cells were the first layer of cells at the border of the islets. Any other cells inside the first layer were considered as central cells. Figure 5 is presented with emphasis on the cells in the center and periphery of islets.

Hypothalamus, duodenum, and stomach sections (Figure 4) imaged at 1.250 µM resolution were counted at their ventromedial of hypothalamus or epithelial region for enteroendocrine cells labelled with SST antibodies in the duodenum and stomach, respectively. The images were normalized by area of the image frame.

### 4.7. Assay Development

Static batch incubation of human or mouse pancreatic islets was conducted by dispensing them in cell culture dishes in groups of 10 or 20 islets in 200 μL of Krebs Ringer buffer (KRB). Islets were incubated at low or high glucose containing KRB for 1 h. Supernatant was collected for somatostatin measurement. For total content, islets were sonicated for 20 s for total lysis of the cells.

A standard curve was made with Somatostatin-14 purchased from Tocris cat# 1157. Antigen coating was performed by adding 150 μL of each standard (in triplicates) or sample to wells of a 96-well high-binding plate from biomat cat# MG01F-HB8. The plate was kept at 4 °C overnight. Wells were washed once with washing buffer and 150 μL blocking buffer was added and once more kept overnight at 4 °C. On day 3, primary antibody SST10G5 Mercodia AB was added at a 1:200 dilution for two hours. Anti-mouse conjugated HRP antibody was then added at a 1:100 dilution for one hour. The wells were washed 3 times with washing buffer and 200 TMB substrate was added. The plate was allowed to develop for 30 min and 50 μL stop solution was added to each well. Absorbance was read at 450 nm maximum 30 min after reaction stop. The assay was performed in 3 replicates. The data represent the average of the 3 replicates. The optical density (OD) of the samples was interpolated in the standard curve using the four parametric logistic curves fit.

### 4.8. RNA Extraction and Quantitative PCR

Total RNA was prepared using Trizol method. Cells were treated with Trizol, then isolated the RNA according to manufacture protocol. The quality and quantity of RNA were checked using nanodrop. The first strand of cDNA synthesis was performed using TAKARA cDNA synthesis kit. Real time (RT-PCR) was performed with a TAKARA SYBR GREEN SUPERMIX according to the manufacturer’s protocol. The final reaction volume was 20 uL. RT-PCR data were analyzed with BIO-Rad IQTM optical system software. Expression levels of targeted genes were compared with internal control.

### 4.9. Statistics 

Data are presented as mean ± s.e.m, unless otherwise stated. Statistical significance was assessed using Students *t*-test for two-tailed, paired, or unpaired samples, as appropriate. Significant difference is indicated by asterisks (* *p* < 0.05, ** *p* < 0.01, *** *p* < 0.001).

## Figures and Tables

**Table 1:** Table documenting the details of the antibodies used.

S. No.	Antibody	Species	Concentration	Monoclonal/Polyclonal	Antibody Against
1	SST14/28 from Immunostar	Rabbit	15 μg/μL	Polyclonal	SST14/28
2	SST14 from Abcam	Rabbit	10 mg/mL	Polyclonal	SST14
3	SST41B4 from Mercodia	Rat	0.4 mg/mL	Monoclonal	SST28
4	SST13D12 from Mercodia	Mouse	0.8 mg/mL	Monoclonal	SST14
5	SST31G12 from Mercodia	Rat	0.7 mg/mL	Monoclonal	SST28
6	SST10G5 from Mercodia	Mouse	0.4 mg/mL	Monoclonal	SST14
7	SST12E7 from Mercodia	Mouse	0.7 mg/mL	Monoclonal	SST14
8	SST32A1 from Mercodia	Rat	0.4 mg/mL	Monoclonal	SST28

## Data Availability

The data will be provided on request. Data will be made available upon requests after approvals from Mercodia AB and all authors.
